# The Tumor Necrosis Factor Superfamily Members TNFSF14 (LIGHT), Lymphotoxin β and Lymphotoxin β Receptor Interact to Regulate Intestinal Inflammation

**DOI:** 10.3389/fimmu.2018.02585

**Published:** 2018-11-22

**Authors:** Daniel A. Giles, Sonja Zahner, Petra Krause, Esmé Van Der Gracht, Thomas Riffelmacher, Venetia Morris, Alexei Tumanov, Mitchell Kronenberg

**Affiliations:** ^1^Division of Developmental Immunology, La Jolla Institute for Allergy and Immunology, La Jolla, CA, United States; ^2^Department of Microbiology, Immunology and Molecular Genetics, University of Texas Health Science Center San Antonio, San Antonio, TX, United States

**Keywords:** TNF superfamily, Colitis, Lymphotoxin (LT), Light, DSS (dextran sulfate sodium)

## Abstract

Over 1.5 million individuals in the United States are afflicted with inflammatory bowel disease (IBD). While the progression of IBD is multifactorial, chronic, unresolved inflammation certainly plays a key role. Additionally, while multiple immune mediators have been shown to affect pathogenesis, a comprehensive understanding of disease progression is lacking. Previous work has demonstrated that a member of the TNF superfamily, TNFSF14 (LIGHT), which is pro-inflammatory in several contexts, surprisingly plays an important role in protection from inflammation in mouse models of colitis, with LIGHT deficient mice having more severe disease pathogenesis. However, LIGHT is a single member of a complex signaling network. It signals through multiple receptors, including herpes virus entry mediator (HVEM) and lymphotoxin beta receptor (LTβR); these two receptors in turn can bind to other ligands. It remains unknown which receptors and competing ligands can mediate or counteract the outcome of LIGHT-signaling during colitis. Here we demonstrate that LIGHT signaling through LTβR, rather than HVEM, plays a critical role in the progression of DSS-induced colitis, as LTβR deficient mice exhibit a more severe disease phenotype. Further, mice deficient in LTαβ do not exhibit differential colitis progression compared to WT mice. However, deletion of both LIGHT and LTαβ, but not deletion of both LTαβ and LTβR, resulted in a reversal of the adverse effects associated with the loss of LIGHT. In sum, the LIGHT/LTαβ/LTβR signaling network contributes to DSS colitis, but there may be additional receptors or indirect effects, and therefore, the relationships between these receptors and ligands remains enigmatic.

## Introduction

Inflammatory bowel disease is an immune-mediated disease in which, among other components, the microbiome, genetics and immune system all contribute to disease (1). Multitudes of bacteria and other microbes reside in the intestine, and at steady state homeostasis is maintained by a controlled and balanced intestinal mucosal immune system (2). This immune system includes various types of epithelial cells, myeloid cells and lymphocytes, along with a plethora of antimicrobial peptides and inflammatory and regulatory mediators that these cells actively produce (3). While the initial driving forces may vary, an imbalance in this immune response can lead to the development of IBD (4). Of interest, different mediators of the mucosal immune system can either protect from, or exacerbate disease (1). Thus, our understanding of the role of different mediators during IBD is evolving.

The tumor necrosis factor (TNF) superfamily of cytokines and receptors have a diverse, but not fully defined, function in mucosal immunity and IBD pathogenesis (5–7). In fact, antibodies blocking TNF are commonly used as therapeutic agents for IBD patients (8). Over expression of TNF superfamily member 14 (TNFSF14, or LIGHT [homologous to **l**ymphotoxins, exhibits **i**nducible expression, and competes with HSV **g**lycoprotein D for **H**VEM, a receptor expressed by **T** cells]) in transgenic mice leads to colitis (9, 10). Also, LIGHT expression by T cells is increased in Crohn's disease patients (11), and LIGHT promotes inflammation in the skin and lung (12, 13). On the other hand, our previous work has shown that LIGHT, surprisingly, exhibits a protective effect in colitis induced by dextran sulfate sodium (DSS) and by transfer of naïve CD4^+^ T cells to immune deficient mice (5). This was most thoroughly studied in the DSS model, in which mice deficient in LIGHT had decreased colon length, increased pathology scores and increased immune cell infiltration to the colonic lamina propria in a chronic DSS model, in which at least two rounds of the chemical were administered. However, whether other members of LIGHT's signaling network affect the progression of DSS-induced colitis remains to be determined.

LIGHT can bind two receptors, the lymphotoxin beta receptor (LTβR or TNFRSF3) and the herpes virus entry mediator (HVEM or TNFRSF14) (14). Both receptors are members of the TNF receptor superfamily. Accordingly, stimulation of either receptor has been previously shown to drive an inflammatory response (14). However, in the context of DSS-induced colitis, these two receptors may have different effects. While LIGHT appears to be protective during DSS-induced colitis (5), HVEM deficient mice exhibit disease pathology similar to wild type (WT) mice (6). Conversely, antibody mediated blockade of LTβR results in worsened colitis (5). The results from these experiments suggest that LTβR is the critical receptor in maintaining the protective effect of LIGHT during DSS-induced colitis. Interestingly, in addition to LIGHT, LTβR can also be activated by surface lymphotoxin (LTαβ), a heterotrimer comprised of two TNF superfamily members, a single unit of TNFSF1 (LTα) and two units TNFSF3 (LTβ) (15). Signaling of LTβR by LTαβ is required for the formation of lymph nodes (16). Additionally, activation of LTβR by LTαβ has also been shown to play a role in a variety of inflammatory disorders (17), but whether LTαβ signaling through LTβR plays a role in intestinal inflammation remains undefined.

Here, we report the generation and analysis of a variety of double-mutant mice to delineate the complex interplay of LIGHT/LTαβ with LTβR/HVEM signaling during DSS-induced colitis. We demonstrate that LIGHT signaling through LTβR is indispensable for protection from exacerbated DSS-induced colitis. Additionally, HVEM activation does not seem to contribute to DSS-induced colitis, even in the absence of LTβR. While LTαβ signaling by itself is not critical for altering the severity of colitis, LTαβ deletion rescued the pathogenic effect of LIGHT deletion, but not of LTβR deletion. This shows that the role of specific ligands becomes difficult to predict when multiple members of the TNF superfamily are depleted, and suggests the possibility that LTαβ has effects that extend beyond its interaction with LTβR, or alternatively, that LTβR integrates additional signals to affect the outcome in DSS-colitis.

## Results

### Lymphotoxin beta receptor activation prevents exacerbated colitis

**Figure 1:**
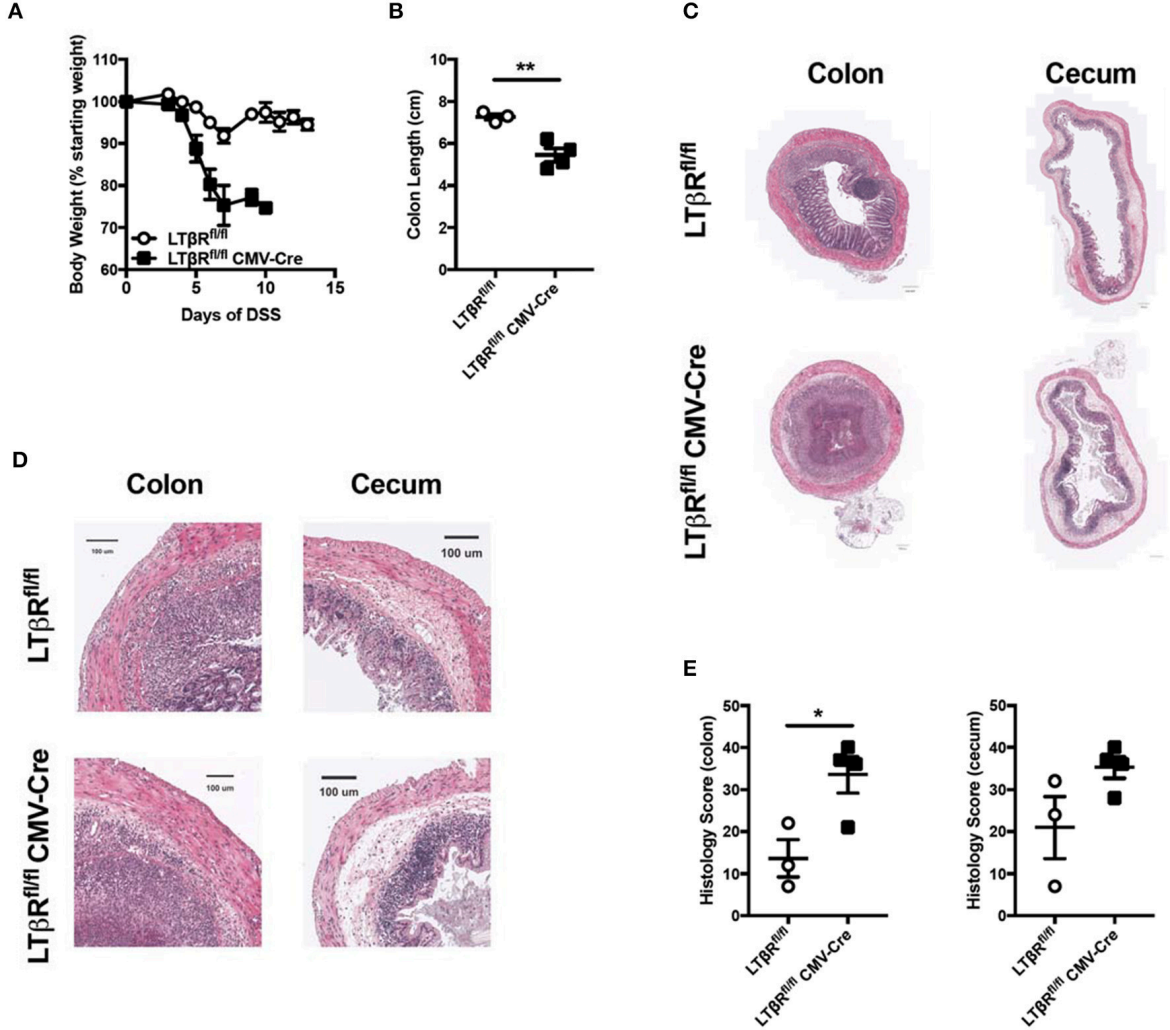
Loss of LTβR leads to exacerbated DSS-induced colitis. Eight week-old *Ltbr*^*fl*/*fl*^ CMV-cre (*n* = 4) and *Ltbr*^*fl*/*fl*^ (*n* = 3) female mice received 3% DSS in the drinking water. **(A)** Weight loss was monitored daily. **(B)** Following termination of experiment, colon lengths were measured. **(C)** Representative H&E staining of cecum and distal colon cross-section (scale bar = 200 μM). **(D)** Magnified section of cecum and distal colon cross-section from **C** (scale bar = 100 μM). **(E)** Histologic scoring of ceca and distal colons. Data are representative of one of three individual experiments. Data represent mean ± S.E.M. Student's *t*-test, *p < 0.05, **p < 0.01.

Previous results demonstrated that LIGHT signaling protects from DSS-induced colitis (5). In the absence of LIGHT, the innate immune response was augmented, especially in the chronic DSS model, with increased IL-6, IL-1β, and oncostatin M (5). Further, one of LIGHT's receptors, HVEM, was not found to contribute to colitis, when mice deficient for HVEM expression were tested. Additionally, an antibody that blocks LIGHT-LTβR but not LTαβ-LTβR binding led to more severe DSS-induced colitis, strongly implicating a role for the LTβR (6). However, the antibody epitope and how it selectively blocked one TNFSF ligand and not the other remains undefined, and it is possible that the antibody has mixed agonist-antagonist properties. Therefore, to more definitively address whether LTβR contributes to LIGHT mediated protection from DSS-induced colitis, we administered DSS in drinking water to LTβR deficient mice and controls. Of note, gene knockout mice were created by crossing *Ltbr*^*fl*/*fl*^ mice to CMV-cre for ubiquitous depletion of LTβR (18, 19). DSS administration resulted in increased weight loss in *Ltbr*^*fl*/*fl*^-CMV-cre mice, compared to controls, indicative of increased disease (Figure 1A) (20). Additionally, *Ltbr*^*fl*/*fl*^-CMV-cre mice displayed a decreased colon length (Figure 1B), typically indicative of fibrosis and a more severe colitis phenotype (20). Indeed, histological analysis of both the colon and cecum of *Ltbr*^*fl*/*fl*^-CMV-cre mice revealed an increased histological score (Figures 1C–E) (5), indicating that DSS-induced colitis is more severe in these mice. Similar to mice deficient for LIGHT protein, mice lacking LTβR had increased inflammatory cell infiltrates, epithelial disruption and evidence for intestinal edema. Additionally, these mice had increased mRNA encoding IL-1β, similar to mice lacking LIGHT (data not shown). Together, these results demonstrate that LTβR activation is necessary for protection from exacerbated DSS-induced colitis, with a phenotype similar to the absence of LIGHT.

### Lymphotoxin signaling does not contribute to colitis progression

**Figure 2:**
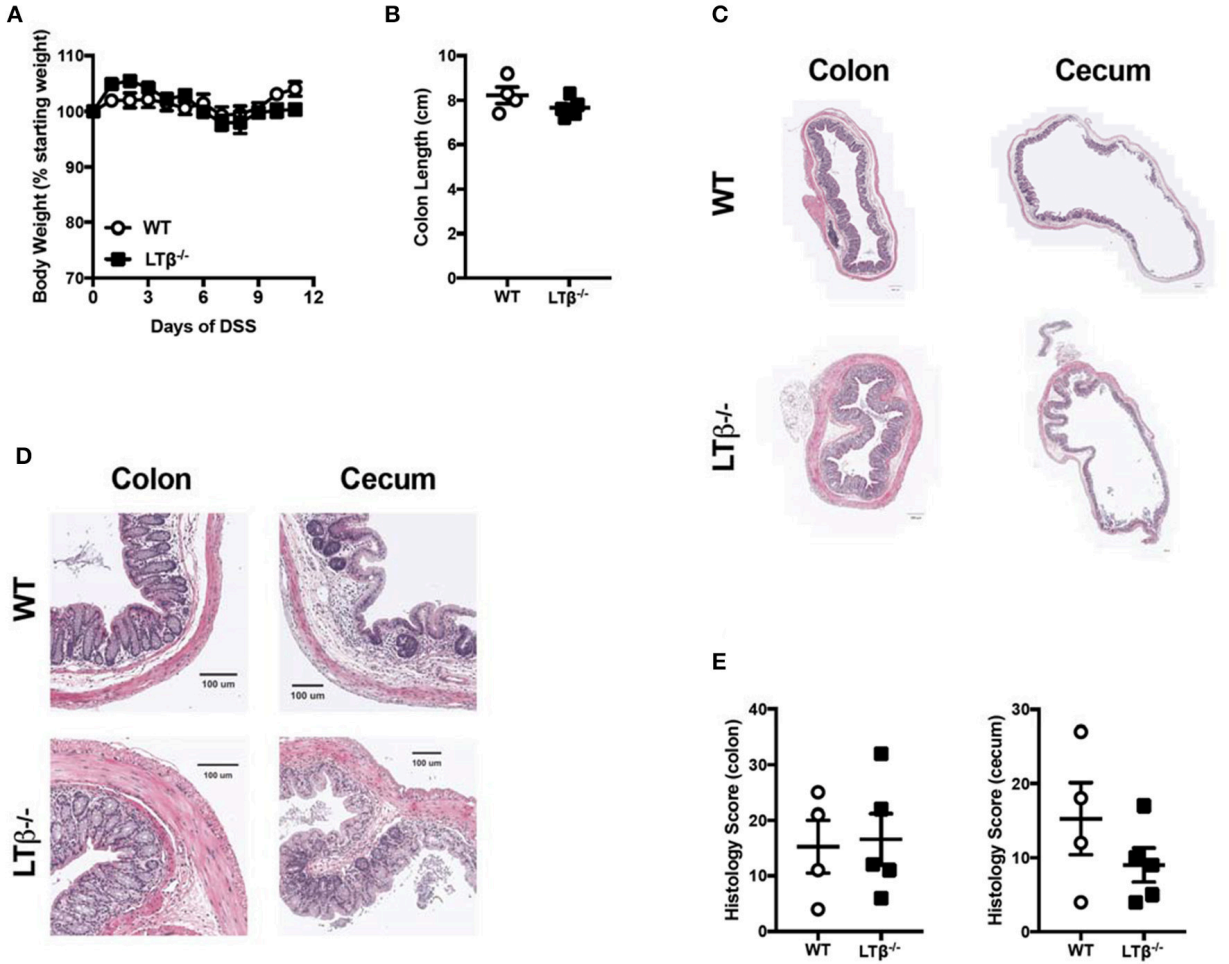
Loss of LTβ does not affect the pathogenesis of DSS-induced colitis. Eight week-old *Ltb*^−/−^ (*n* = 5) and WT (*n* = 4) female mice received 2% DSS in the drinking water. **(A)** Weight loss was monitored daily. **(B)** Following termination of experiment, colon lengths were measured. **(C)** Representative H&E staining of cecum and distal colon cross-section (scale bar = 200 μM). **(D)** Magnified section of cecum and distal colon cross-section from **C** (scale bar = 100 μM). **(E)** Histologic scoring of ceca and distal colons. Data are representative of one of three individual experiments. Data represent mean ± S.E.M. Student's *t*-test.

In addition to activation via LIGHT, LTβR can also be activated by a second ligand, LTαβ (15). While LIGHT can be both soluble and a cell surface protein, LTαβ is exclusively a cell-surface heterotrimer comprised of one LTα unit two LTβ units. Notably, LTβ is required for LTα to bind LTβR, so in LTβ deficient mice the only available signaling through LTβR is via LIGHT (15). Thus, to determine whether LTαβ contributes to LTβR protective effects in in DSS-induced colitis, LTβ deficient mice were treated with DSS. Unlike LTβR deficient and LIGHT deficient mice, *Ltb*^−/−^ mice exhibited weight loss and colon lengths similar to WT controls after DSS treatment (Figures 2A,B). Further, histological analysis revealed that *Ltb*^−/−^ mice also exhibited a phenotype similar to WT controls (Figures 2C–E). These data demonstrate that LTαβ signaling through LTβR does not contribute to preventing severe DSS-induced colitis, consistent with the hypothesis that LIGHT-LTβR binding is essential.

### Mice deficient in both light and LTβ are protected from exacerbated colitis

**Figure 3:**
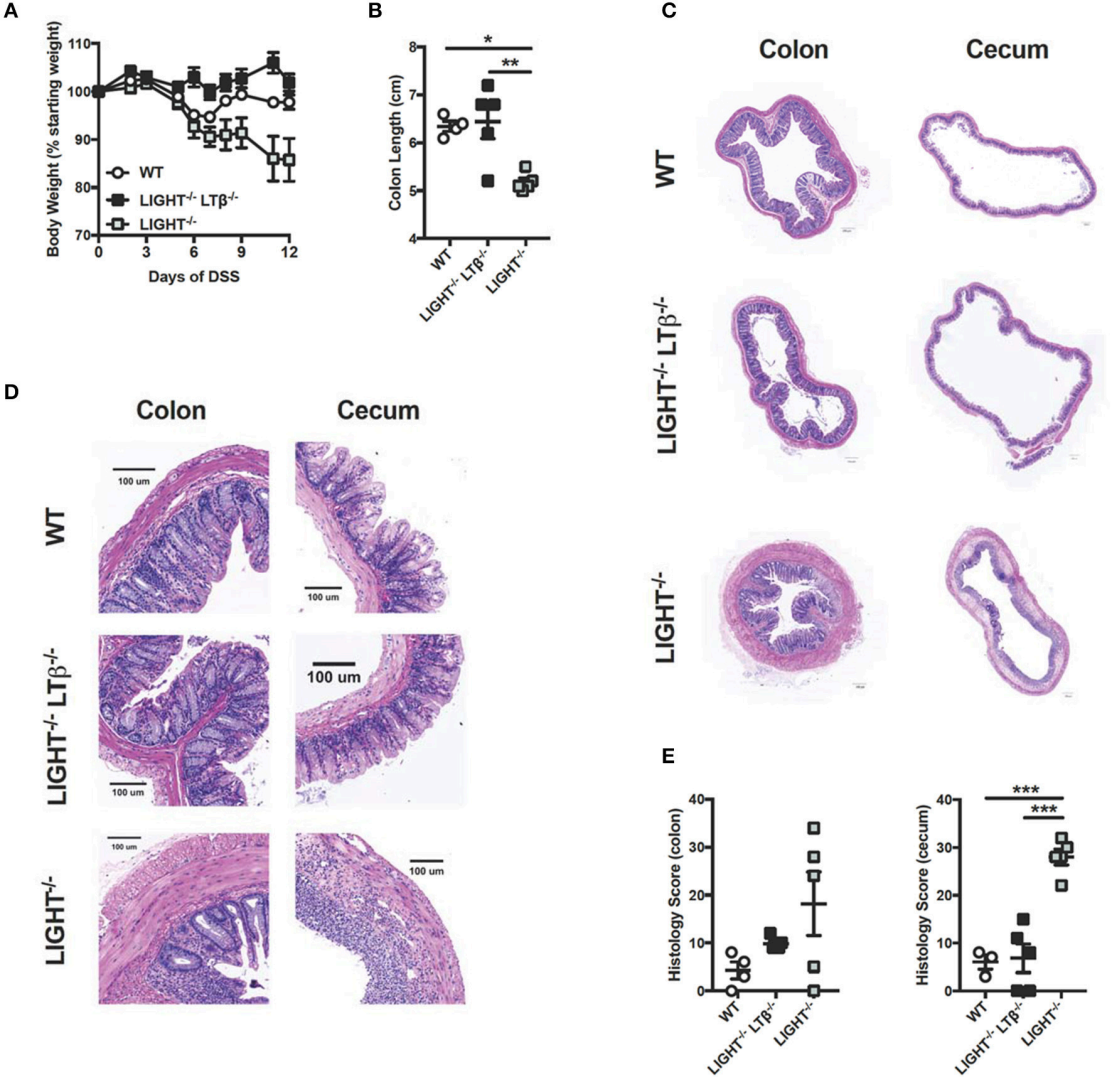
Combined deficiency of LTβ and LIGHT rescues from severe colitis. Eight week-old *Light*^−/−^*Ltb*^−/−^ (*n* = 5), *Light*^−/−^ (*n* = 5) and WT (*n* = 4) male mice received 3% DSS in the drinking water. **(A)** Weight loss was monitored daily. **(B)** Following termination of experiment, colon lengths were measured. **(C)** Representative H&E staining of cecum and distal colon cross-section (scale bar = 200 μM). **(D)** Magnified section of cecum and distal colon cross-section from **C** (scale bar = 100 μM). **(E)** Histologic scoring of ceca and distal colons. Data represent mean ± S.E.M. One way ANOVA with Tukey's correction, *p < 0.05, ***p < 0.001.

Given that LIGHT protects from exacerbated DSS-induced colitis and that LTαβ does not contribute to enhanced colitis progression, we hypothesized that mice deficient for both LIGHT and LTβ would develop augmented colitis. To test whether the absence of both TNFSF cytokines would affect colitis progression, DSS was administered to *Light*^−/−^*Ltb*^−/−^ mice and WT controls. After 12 days, the effects of DSS administration were evaluated. Unlike *Light*^−/−^ mice, which displayed a more rapid weight loss, *Light*^−/−^*Ltb*^−/−^ mice exhibited little weight loss and had colon lengths similar to controls (Figures 3A,B). Further, *Light*^−/−^*Ltb*^−/−^ colons and cecal tissue appeared similar to those of DSS-treated WT controls, while the colon and cecum *Light*^−/−^ mice displayed increased inflammation, quantified by an increased histological score (Figures 3C–E). These results indicate that deficiency of LIGHT is not sufficient to exacerbate DSS-induced colitis when LTβR signaling by LTαβ is also impaired. Additionally, these observations confound our understanding of this signaling network in DSS and suggest that other mechanisms may be contributing. One possible explanation is that in the absence of LIGHT signaling, LTαβ binds to LTβR and drives increased inflammation.

### Mice deficient in both LTβ and LTβR exhibit exacerbated colitis

**Figure 4:**
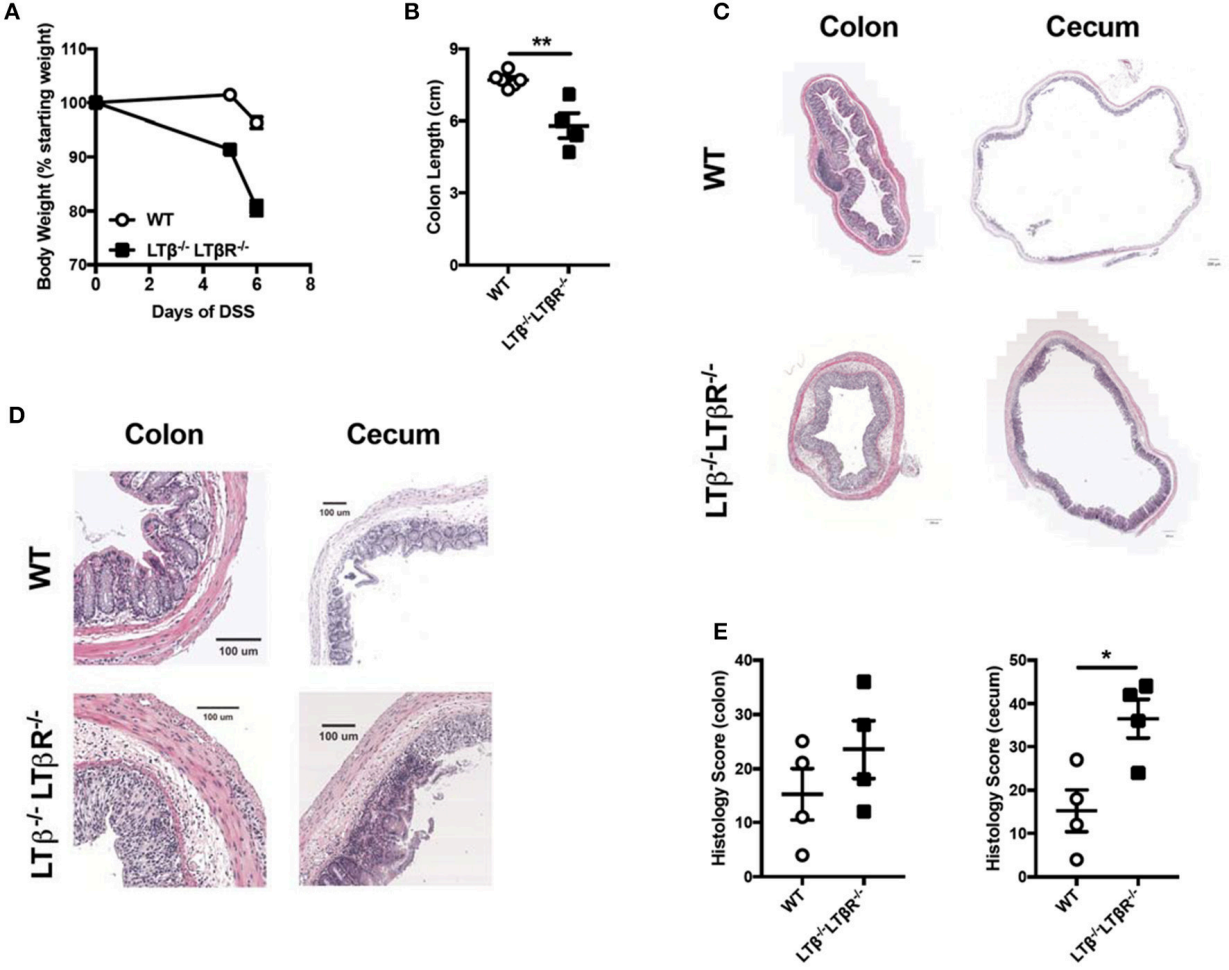
Combined deficiency of LTβ and LTβR causes exacerbated DSS-induced colitis. Eight week-old *Ltb*^−/−^*Ltbr*^−/−^ (*n* = 4) and WT (*n* = 4) female mice received 2% DSS in the drinking water. **(A)** Weight loss was monitored daily. **(B)** Following termination of experiment, colon lengths were measured. **(C)** Representative H&E staining of cecum and distal colon cross-section (scale bar = 200 μM). **(D)** Magnified section of cecum and distal colon cross-scetion from **C** (scale bar = 100 μM). **(E)** Histological scoring of ceca and distal colons. Data are representative of one of two individual experiments. Data represent mean ± S.E.M. Student's *t*-test, *p < 0.05, **p < 0.001.

To directly test the hypothesis that LTβ-LTβR signals drive severe colitis, we crossed two strains to generate double knock out (DKO) mice deficient for LTαβ and LTβR and determined if these mice exhibited augmented DSS-induced colitis progression. In these DKO mice, LIGHT-HVEM interactions occur independently of a possible HVEM competition with LTβR for binding to this ligand. Administration of DSS to *Ltb*^−/−^*Ltbr*^−/−^ mice resulted increased weight loss compared to WT controls (Figure 4A). This increased weight loss correlated with decreased colon length in *Ltb*^−/−^*Ltbr*^−/−^ mice (Figure 4B). Further, histological analysis of the colon and cecum revealed that *Ltb*^−/−^*Ltbr*^−/−^ mice exhibited an increased histology score, indicative of increased inflammation in the tissue (Figures 4C–E). The exacerbation of colitis in the combined absence of LTαβ and LTβR disproves the hypothesis that in the absence of LIGHT increased binding of LTβ to LTβR drives disease.

### Mice deficient in HVEM and LTβR exhibit exacerbated colitis

**Figure 5:**
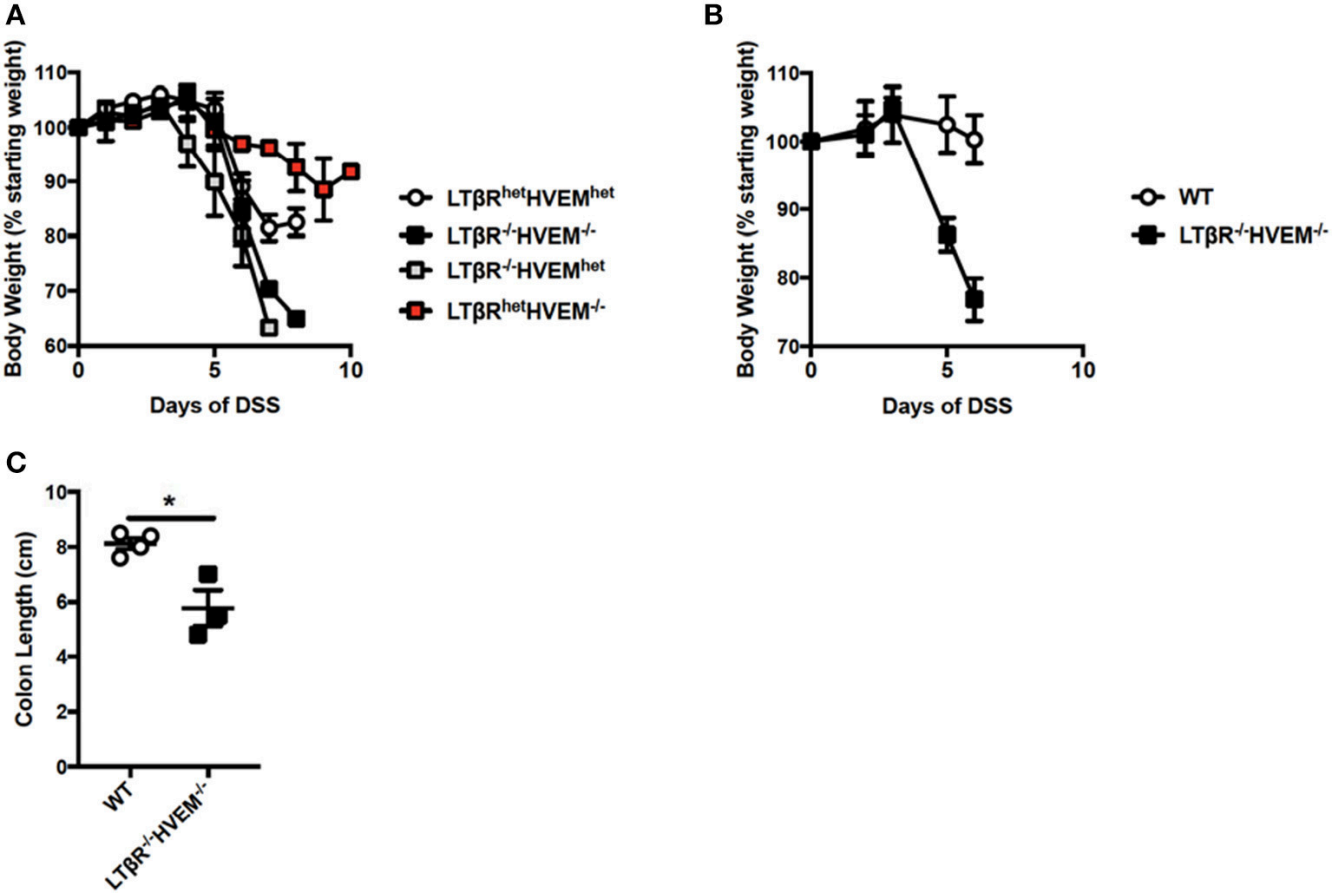
Severe colitis in mice deficient for LTβR and HVEM. **(A)** Eight week-old *Ltbr*^−/−^*Hvem*^−/−^ (*n* = 3), *Ltbr*^*het*^*Hvem*^−/−^ (*n* = 3), *Ltbr*^−/−^*Hvem*^*het*^ (*n* = 3) and *Ltbr*^*he*−^*Hvem*^*het*^ (*n* = 4) male mice received 3% DSS in the drinking water. Weight loss was monitored daily. **(B,C)** Eight week-old *Ltbr*^−/−^*Hvem*^−/−^ (*n* = 3) and WT (*n* = 4) control female mice received 2.5% DSS in the drinking water. **(B)** Weight loss was monitored daily. **(C)** Following termination of experiment, colon lengths were measured. Data represent mean ± S.E.M. Student's *t*-test, *p < 0.05.

It is possible that LTβR and HVEM compete for LIGHT and that in the absence of LTβR, LIGHT binding to HVEM drives inflammation. In this proposed mechanism, LTβR acts in part as a sink for LIGHT protein, preventing it from binding HVEM to the fullest extent. To test this mechanism, we analyzed DSS colitis in *Ltbr*^−/−^*Hvem*^−/−^ DKO mice. Notably, if HVEM signals drive severe inflammation in the absence of LTβR, mice deficient in both receptors should be protected from exacerbated DSS-induced colitis. However, after administration of DSS, *Ltbr*^−/−^*Hvem*^−/−^ mice exhibited increased weight loss after DSS treatment, similar to *Ltbr*^−/−^*Hvem*^*het*^^(+/−)^ mice (Figure 5A). *Ltbr*^*het*^*Hvem*^−/−^ mice displayed weight loss similar to *Ltbr*^*het*^*Hvem*^*het*^ mice. In addition to increased weight loss compared to LTβR^het^HVEM^het^ mice, subsequent studies found that *Ltbr*^−/−^*Hvem*^−/−^ mice also exhibited increased weight loss compared to WT mice (Figure 5B). Additionally, this increased weight loss correlated with shorter colon lengths (Figure 5C). In sum, these data suggest that LIGHT does not signal through HVEM to drive severe inflammation in the absence of LTβR.

## Discussion

Inflammatory bowel disease affects over 1.5 million Americans, and effective treatment options for this debilitating autoimmune disorder are lacking (21). While anti-TNF therapies have proved efficacious in certain patient populations, more therapeutic approaches are clearly needed (22). Given that other members of the TNF superfamily have been shown to play a role in a mouse model of colitis, we aimed to interrogate the LIGHT/LTαβ/LTβR/HVEM signaling network to determine if one or more of the involved components displays an important role in DSS-induced colitis pathogenesis. Analysis of this signaling cascade revealed a complex interaction between ligands and receptors that is influenced by which members are present.

Our previous work demonstrated that deficiency of LIGHT leads to exacerbated DSS-driven colitis (5). This suggested that LIGHT plays a protective role, directly or indirectly, in the context of colitis pathogenesis. Herein, we demonstrate that the likely LIGHT binding receptor for this phenomenon is LTβR. Similar to LIGHT deficient mice, genetic ablation of LTβR resulted in exacerbated colitis with a similar overall phenotype, consistent with previous reports (23, 24). Conversely, removal of the other LIGHT receptor, HVEM, had no effect on DSS-induced colitis, even in the absence of LTβR (Figure 5). It remains to be determined which cells in the colon are critical for LIGHT and LTβR expression. Determining the critical LIGHT expressing cell type in disease models has proven difficult given that antibodies reactive for mouse LIGHT are of insufficient quality. On the other hand, it is well known that epithelial, stromal and myeloid cells express LTβR, but lymphocytes do not (5, 18, 25). We found that LTβR mRNA is expressed by fibroblasts, neutrophils and other CD11b^+^ cells at steady-state and during DSS-induced colitis (5). Examining cell type specific LTβR knockouts in the context of disease could aid in identifying which cell type(s) is most important and help to improve our understanding of the mechanisms underlying severe disease. Thus, although the full mechanism remains elusive, our data strongly suggest that signaling of LTβR via LIGHT is necessary for protection from exacerbated DSS-induced colitis.

On the other hand, *Ltb*^−/−^ and *Light*^−/−^*Ltb*^−/−^ mice are protected from exacerbated colitis pathogenesis. The fact that *Ltb*^−/−^ mice do not display differential colitis pathogenesis led to our initial belief that LTαβ does not contribute to DSS-induced colitis. However, if this were the case then *Light*^−/−^*Ltb*^−/−^ mice should show a similar phenotype to *Light*^−/−^mice, which is not the case. This could indicate that in the absence of LIGHT, LTαβ drives inflammation through LTβR. However, as demonstrated in Figure 4, *Ltb*^−/−^*Ltbr*^−/−^ mice exhibited exacerbated colitis, which opposes this hypothesis.

The fundamental conundrum is that mice deficient for LTβR expression have a different phenotype in DSS colitis from mice deficient for both of its known ligands, LIGHT and LTβ. This cannot be explained by a compensating effect of increased signaling by LIGHT-HVEM when LTβR is missing (Figure 5). We cannot rule out the possibility of an indirect effect, such that when *Ltb* is deleted there is increased LTα3 expression. This cytokine can signal through both TNFR1 and TNFR2, and the increased signaling could be protective. It is uncertain, however, why increased LTα3 would be protective in the context of LIGHT deficiency but not LTβR deficiency. It is also possible that there is another player in this signaling network, either an additional receptor for LTβ or another ligand for LTβR. Recent findings have indicated that some TNFSF receptors have multiple ligands, including HVEM and 4-1BB (26), including binding partners for these receptors that are not TNFSF proteins. In this regard, it is of interest that LTβR deficiency has a greater effect on lymph node genesis than either LTβ deficiency or LIGHT deficiency, suggesting LTβR might integrate other signals. We note that the absence of LIGHT has little or no effect on lymph node genesis, in the absence of LTβ caudal and mesenteric lymph nodes are still present, while all lymph nodes require LTβR (14). Additionally, we cannot rule out a technical issue in comparing different gene deficient strains, such as an effect of a gene deletion on a nearby gene or the presence of a few non-C57BL/6 genes remaining in one of the strains not created on the C57BL/6 background, despite extensive back crossing. In sum, the LIGHT/LTβR signaling critically contributes to DSS-induced colitis, but is subject to a degree of opposing regulation in the absence of LTαβ. Further work is needed to fully delineate this signaling network and how it affects intestinal disease in a cell-type specific manner.

## Methods

### Animals

All mice were bred and housed under specific pathogen-free conditions at the La Jolla Institute for Allergy and Immunology (La Jolla, CA). All mice were on the C57BL/6J background. C57BL/6J were originally purchased from the Jackson Laboratory. HVEM mice were bred and described previously (27). *Ltbr*^−/−^ mice were generated by crossing mice with a CMV-cre construct (Jackson Laboratories; Bar Harbor, ME) to *Ltrb*^*fl*/*fl*^ mice, that were previously described (18). LIGHT deficient mice (*Tnfsf14*^−/−^) and *Ltb*^−/−^ mice were provided by Dr. Klaus Pfeffer (University of Düsseldorf, Germany) (28). Double mutants were created by inter-crossing of the above strains. All procedures were approved by the La Jolla Institute for Allergy and Immunology Animal Care and Use Committee.

### Chronic dextran sulfate sodium-induced colitis

Mice received 2.5% DSS (Affymetrix) in the drinking water for a maximum of two cycles. As previously described, 1 cycle is comprised of 5 days of water plus DSS and 2 days with regular drinking water without DSS (29). Given that both male and female mice develop robust colitis after DSS administration (30), both sexes were used for separate experiments but never mixed, as noted in the figure legends. Body weight and appearance were monitored daily. Mice were euthanized in compliance with our animal protocols within 24 h of losing more than 20% of their starting body weight.

### Histology

Upon termination of an experiment, cecum and colon were isolated. Following measurement of colon length, a piece of distal colon and cecum were fixed in zinc formalin (Medical Chemical Corporation). Following paraffin embedding, fixed tissue was stained with hematoxylin and eosin. Resulting slides were then blinded and scored according to previously described criteria (5). Representative images were selected from 5 or more sections per organ, generated on an Axioscan Z1 platform (Zeiss) with a 40x objective in automatic scan mode and Zeiss Zen 2.3 software. Scale bars represent 200 μm for cross-sections and 100 μm for magnified images.

### Statistical methods

All data were analyzed using GraphPad Prism 7 software. Statistical significance was determined by unpaired Student's *t*-test for direct comparisons when there were two groups. For determination of statistical significance for three or more groups, one-way ANOVA was employed with Tukey's *post hoc* test to assess differences between specific groups. All data are displayed as mean with standard error of the mean (S.E.M.).

## Author contributions

MK and AT contributed to the design of the study. DG, SZ, PK, EV, TR, and VM performed the experiments. DG, SZ, PK, and MK analyzed the data. DG and MK drafted the manuscript.

### Conflict of interest statement

The authors declare that the research was conducted in the absence of any commercial or financial relationships that could be construed as a potential conflict of interest.
